# Disease recurrence in a transplant kidney in a patient with extramedullary plasmacytoma

**DOI:** 10.4103/0971-5851.73600

**Published:** 2010

**Authors:** Pooja Binnani, M. M. Bahadur, Nikhil Kedia, Prakash Sharbidre

**Affiliations:** *Department of Nephrology, Jaslok Hospital and Research Centre, Mumbai, India*

**Keywords:** *Cast nephropathy*, *disease recurrence*, *plasmacytoma*

## Abstract

Renal transplantation in patients with malignancy is controversial. Renal transplantation is generally not considered for patients with multiple myeloma (MM) because of their extremely poor prognosis. There are few reports of MM recurrence among kidney transplant recipients. We present a case of disease relapse of plasmacytoma in a transplanted kidney. We present a patient with extramedullary plasmacytoma, who responded well to chemotherapy and underwent allogenic renal transplantation. He relapsed after 4 years with progression to extramedullary plasmacytoma. Despite minimal clinical symptoms, the patient had developed myeloma cast nephropathy and acute renal failure. His renal failure settled after excision of tumor. Extramedullary plasmacytoma as a mode of relapse is highly unusual. Experience of renal transplantation in MM is limited. In the literature, the recurrence of MM is mentioned as a severe complication with a poor graft prognosis. Extramedullary plasmacytoma as a mode of relapse is highly unusual. It should not be considered as a contraindication for transplantation. Renal transplantation for patients with end stage renal disease (ESRD) due to MM is possible. But large prospective studies are needed to develop a strategy for preventing multiple myeloma recurrence.

## INTRODUCTION

Renal transplantation in patients with multiple myeloma (MM) is generally not considered because of extremely poor prognosis. There are few reports of MM recurrence among kidney transplant recipients. We present a patient with plasmacytoma, who responded well initially to chemotherapy and underwent allogenic renal transplantation, but relapsed after 4 years and developed cast nephropathy in the transplant kidney. His renal failure settled after excision of tumor.

## CASE REPORT

A 38-year-old male, with no preexisting diseases like diabetes, hypertension or any past or family history of renal disease, presented in June 2001 with a 4-month history of bilateral loin pain, nocturia, weight loss and feeling unwell. He was pale with periorbital edema and had tenderness in his left loin. No other abnormal features were noted. Investigations showed the following: hemoglobin 6.7 g/dl; leukocytes 6800/d1; platelets 169,000/dl; sternal marrow showed active hemopoiesis with normal plasma cells; urea 196 mg/dl; serum creatinine 12.3 mg/dl; serum potassium 6.3 mEq/l; serum albumin 3.4 g/l; 24-hour urine output 150 ml. Serum electrophoresis was normal but immunoelectrophoresis of plasma and urine showed free kappa light chains. There was mild immunoparesis: IgG 810 mg/dl; IgM 64 mg/dl; IgA 63 mg/dl (normal range: IgG 560–1800 mg/dl; IgM 45–250 mg/dl; IgA 100–400 mg/dl). Ultrasonography showed a soft tissue left lumbar mass and normal sized kidneys. Skeletal survey was non-contributory. On aspiration cytology of the lumbar mass, there was dense plasma cell infiltration with many multinucleate cells. Renal biopsy showed myeloma cast nephropathy with negative congo red stain.

He was maintained on hemodialysis twice weekly. He was treated with four cycles of chemotherapy with VAD (Vincristine 0.4 mg/day, Doxorubicin 9 mg/m^2^/day by continuous intravenous infusion on days 1–4, and Dexamethasone 40 mg/day by infusion on days 1–4, 9–12 and 17–20), followed by Thalidomide 100 mg/daily plus Dexametasone 40 mg/daily for 4 days each month for 6 months. At the end of therapy, the patient was re-assessed. His serum free light assay was unmeasurable and soft tissue mass regressed by more than 50% in size. Thus, his disease went into partial remission and remained stable thereafter, but he remained dialysis-dependent for the next 2 years. Surgery could not be offered to this patient after partial response, as he was lost to follow-up.

In 2003, the patient underwent kidney transplant at another center with his sister as a donor. The patient had one haplotype HLA match. On laprotomy, a left lumbar mass was noted; frozen biopsy of the mass showed absence of malignant cells. Probably, this was the same mass that was noted earlier. The patient received Prednisolone, Cyclosporine and Azathioprine as immunosuppressants. Renal transplantation was uneventful. His serum creatinine was 0.9 mg% on day 15. Post-transplant, the patient was monitored for recurrence of myeloma with physical examination, urine immonofixation and biochemistry including renal function tests and serum calcium. He did well till July 2007 with creatinine remaining between 1 and 1.2 mg/dl.

**Figure 1:**
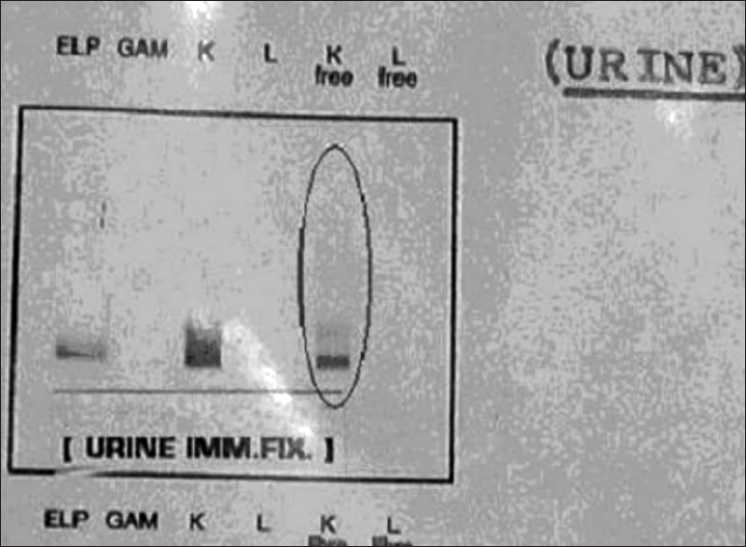
Urine immunofixation showing free kappa light chains

In July 2007, he noticed frothy urine; urine routine showed 4 + proteinuria. Further investigations showed the following: serum creatinine 1.1 mg%; serum calcium 9.4 mg%; serum phosphorus 5 mg%; serum uric acid 7.4 mg%; total protein 7.3 g%; serum albumin 3.6 g%; alkaline phosphatase (ALP) 156 mg%; Serum protein electrophoresis (SPE) and serum protein IF – no M band; Hb 11.3 g%; WBC 7540/dl; platelets 315,000/dl; 24 hour urine protein 1.07 g/2100 ml; bone marrow study – no abnormal cells seen in marrow but urine immunofixation showed free kappa light chains [Figure 1].

The patient was put on Thalidomide + steroid, for 6 months. He developed Thalidomide-induced neuropathy. Urine immunofixation showed increased free kappa light chains and the serum creatinine increased to 3.4 mg%. Repeat bone marrow study showed absence of abnormal cells.

Renal graft biopsy done at the transplant center showed 12 glomeruli displaying normal capillary basement membrane, tubules showing fractured hyaline casts surrounded by mononuclear giant cells along with interstitial edema and marked mononuclear inflammatory cell infiltration. Thus, there was recurrence of myeloma cast nephropathy in renal graft.

He received two cycles of liposomal Doxorubicin (30 mg/m^2^once every 4 weeks). He was admitted on 16 April 2008 for third cycle, but was found to have serum creatinine 8.8 mg%. He was clinically stable, had no features of uremia and fluid overload. He was put on oral Dexamethasone 40 mg OD.

**Figure 2:**
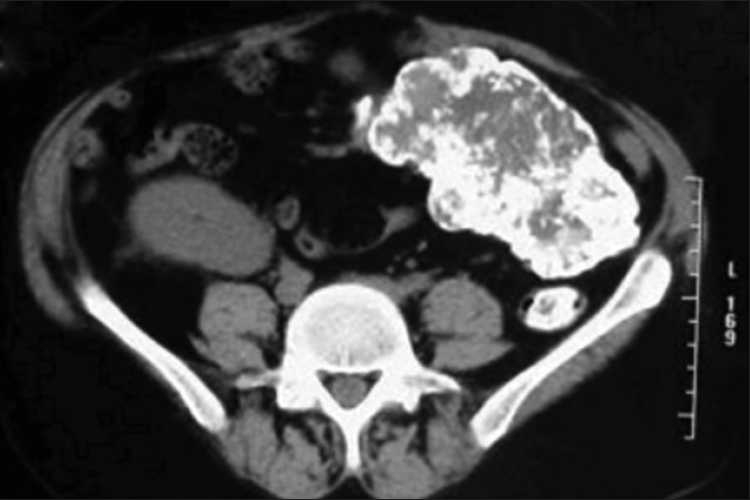
CT scan of abdomen showing, large calcified mass in left lower abdomen with extensive retroperitoneal and mesenteric lymphadenopathy

After giving high dose steroid, serum creatinine reduced to 5.7 mg%. Ultrasonography of abdomen showed a large left lumbar mass. Computed tomography (CT) scan abdomen revealed a large calcified lobulated mass in left lower abdomen and upper pelvis and associated retroperitoneal and mesenteric lymphadenopathy [Figure 2].

Tumor excision was planned. To avoid intraoperative complications, one session of hemodialysis was done a day prior to surgery. Extensive, hard and irregular mass was seen arising from small bowel mesentery extending up to the root of mesentery and engulfing superior mesenteric vein. Histology was suggestive of massive plasmacytoma with huge deposits of myeloma protein (95% tumor made up of dense hyaline, partly proteinaceous and partly fibrillar material). It was covered by well-differentiated monomorphic plasma cells possessing coarse hyperchromatic nuclei. Lymph nodes were unremarkable. Congo red stain was negative.

One month later, his serum creatinine was 2 mg% and BUN Blood urea nitrogen was 45 mg%. Rest of the laboratory tests were normal. Urine showed trace proteins. In urine immunofixation, faint free kappa light chains were detected. At this point, the patient was considered for therapy with Thalidomide and Dexamethasone.

## DISCUSSION

Renal transplantation in most patients with myelomatous diseases is not considered a viable option because of poor prognosis. Data from the Cincinnati Transplant Tumor Registry of 1137 patients, showed recurrences in MM in up to 67% patients.[1] Tumor growth is probably more rapid in these patients because of immunosuppression, poor immune surveillance,[2] susceptibility to viral infection (especially Epstein–Barr virus),[3] chronic antigenic stimulation due to the presence of foreign allograft antigens, cytokines such as IL-6 and IL-10,[4] and direct effect by immunosuppressants like Azathioprine.[5]

There are few reports of MM recurrence among kidney transplant recipients. We present a patient with extramedullary plasmacytoma, who responded well initially to chemotherapy and underwent allogenic renal transplantation, but relapsed after 4 years after a disease free interval, and developed myeloma cast nephropathy. His renal failure settled after excision of tumor.

Extramedullary plasmacytoma with a complicating renal failure is highly unusual. We have been unable to find similar report in the literature.

## CONCLUSION

In this rare case, renal failure because of recurrent cast nephropathy in a renal allograft regressed after excision of the plasmacytoma. Extramedullary plasmacytoma as a mode of relapse is highly unusual. Renal transplantation for patients with end stage kidney disease due to myelomatous diseases is possible but needs large prospective studies to develop a strategy for preventing recurrence.
